# Over-Representation of Somali Patients in an Early Intervention in Psychosis Service in Leicester City: A Service-Level Epidemiological Analysis

**DOI:** 10.1192/bjo.2026.11121

**Published:** 2026-06-30

**Authors:** Yassmine Sabtan, Nandini Chakraborty

**Affiliations:** Leicestershire Partnership Trust, Leicester, United Kingdom

## Abstract

**Aims:**

Psychotic disorders display marked ethnic disparities in incidence in the UK, with Black African and Black Caribbean populations consistently shown to have elevated risk.Leicester City is one of the UK’s most ethnically diverse cities and hosts a large and long-standing Somali community. Clinicians within the Psychosis Intervention and Early Recovery (PIER) service have perceived disproportionate Somali representation. This study quantitatively examines whether Somali patients are over-represented in PIER relative to population size and compares this pattern with African and Caribbean groups.

**Objectives:**

- To determine whether Somali patients are over-represented in the Psychosis Intervention and Early Recovery (PIER) service relative to their proportion in Leicester City’s population.
- To compare Somali over-representation to that of combined Black African/Caribbean groups.
- To quantify crude prevalence, odds ratios (ORs), confidence intervals (CIs), and statistical significance of observed over-representation.

**Methods:**

A retrospective analysis was conducted using a Leicester-City-only EIP caseload dataset (n=310). Ethnicity categories were collapsed into Somali, African/Caribbean (African + Caribbean + Other Black), and Other groups. Population denominators were taken from ONS Census data, including 2150 Somali write-in responses in Leicester City and African/Caribbean proportions based on Black African (~4%) and Black Caribbean (~1.36%) census data. Statistical methods included crude proportions, ORs, 95% CIs, and χ² tests.

**Results:**

Somali patients accounted for 7.7% of the EIP case load but only 0.58% of the Leicester population. OR for Somali over-representation was 13.45, p <0.001. African/Caribbean patients accounted for 10.3% of cases vs 5.36% of the population, OR 1.93, p <0.001. Somali patients were markedly more over-represented than the broader African/Caribbean group.

**Conclusion:**

Somali individuals are substantially over-represented in early psychosis service usage in Leicester. This pattern exceeds the known elevated risk observed in African and Caribbean groups. Findings highlight urgent public health, cultural, and service-access implications requiring further investigation.

